# Clinical and cost-effectiveness of diverse posthospitalisation pathways for COVID-19: a UK evaluation using the PHOSP-COVID cohort

**DOI:** 10.1136/bmjresp-2025-003224

**Published:** 2025-10-31

**Authors:** Andrew H Briggs, Andrew Ibbetson, Archie Walters, Linzy Houchen-Wolloff, Natalie Armstrong, Tristan Emerson, Rhyan Gill, Claire Hastie, Paul Little, Charlotte Overton, John Pimm, Krisnah Poinasamy, Sally Singh, Samantha Walker, Olivia C Leavy, Matthew Richardson, Omer Elneima, Hamish McAuley, Aarti Shikotra, Amisha Singapuri, Marco Sereno, Ruth M Saunders, Victoria C Harris, Neil J Greening, Ewen Harrison, Annemarie Docherty, Nazir I Lone, Jennifer K Quint, James Chalmers, Ling-Pei Ho, Alex Robert Horsley, Betty Raman, Louise V Wain, Christopher E Brightling, Michael Marks, Rachael A Evans, PHOSP-COVID Study Collaborative Group

**Affiliations:** 1 Department of Health Services Research and Policy, London School of Hygiene and Tropical Medicine, London, UK; 2 NIHR Leicester Biomedical Research Centre - Respiratory, Glenfield Hospital, Leicester, UK; 3 Department of Population Health Sciences, University of Leicester, Leicester, UK; 4 PPI Group, BBC Leicester, Leicester, UK; 5 PHOSP-COVID Patient and Public Involvement Group, Leicester NIHR Biomedical Research, Leicester, UK; 6 Long Covid Support, London, UK; 7 Primary Care Research Centre, Faculty of Medicine, University of Southampton, Southampton, UK; 8 Healthy Minds, The Buckinghamshire IAPT Service, Oxford Health NHS Foundation Trust, Oxford, UK; 9 NIHR Oxford Health Biomedical Research Centre, Oxford Health NHS Foundation Trust, Oxford, UK; 10 Asthma + Lung UK, London, UK; 11 Cardiac/Pulmonary Rehabilitation, University Hospitals of Leicester NHS Trust, Leicester, UK; 12 Department of Respiratory Sciences, University of Leicester, Leicester, UK; 13 The Institute for Lung Health, Leicester NIHR Biomedical Research Centre Respiratory, University Hospitals of Leicester NHS Trust, Leicester, UK; 14 Division of Public Health and Epidemiology, School of Medical Sciences, University of Leicester, Leicester, UK; 15 Leicester Respiratory Biomedical Research Unit, National Institute for Health Research, Leicester, UK; 16 NIHR Biomedical Respiratory Centre, University of Leicester, Leicester, UK; 17 The Institute for Lung Health, Leicester NIHR Biomedical Research Centre- Respiratory, University Hospitals of Leicester NHS Trust, Leicester, UK; 18 University Hospitals of Leicester NHS Trust, Leicester, UK; 19 Respiratory, The institute for Lung Health, NIHR Leicester Biomedical Research Centre, University of Leicester, Leicester, UK; 20 Respiratory Medicine, Institute for Lung Health, Leicester, UK; 21 Respiratory Sciences, University of Leicester, Leicester, UK; 22 University of Edinburgh Centre for Medical Informatics, The Usher Institute, Edinburgh, UK; 23 Usher Institute for Population Health Sciences and Informatics, University of Edinburgh, Edinburgh, UK; 24 Royal Infirmary of Edinburgh, NHS Lothian, Edinburgh, UK; 25 School of Public Health, Imperial College, London, UK; 26 Tayside Respiratory Research Group, University of Dundee, Dundee, UK; 27 University of Dundee, Ninewells Hospital and Medical School, Dundee, UK; 28 MRC Human Immunology Unit, Weatherall Institute of Molecular Medicine, Oxford, UK; 29 Oxford Centre for Respiratory Medicine, Churchill Hospital, Oxford, UK; 30 Respiratory Medicine, Manchester University NHS Foundation Trust, Manchester, UK; 31 NIHR Manchester Biomedical Research Centre, Division of Infection, Inflammation and Respiratory Medicine, University of Manchester, Manchester, UK; 32 Division of Cardiovascular Medicine, Radcliffe Department of Medicine, University of Oxford, Oxfordshire, UK; 33 Oxford University Hospitals NHS Foundation Trust, Oxford, UK; 34 Institute of Lung Health, University of Leicester, Leicester, UK; 35 Department of Clinical Research, London School of Hygiene and Tropical Medicine, London, UK; 36 Hospital for Tropical Diseases, University College Hospital London, London, UK

**Keywords:** COVID-19, Health Economist

## Abstract

**Background:**

Long covid has emerged as a complex health condition for millions of people worldwide following the COVID-19 pandemic. Previously, we have categorised healthcare pathways for patients after discharge from hospital with COVID-19 across 45 UK sites. The aim of this work was to estimate the clinical and cost-effectiveness of these pathways.

**Methods:**

We examined prospectively collected data from 1013 patients at 12 months postdischarge on whether they felt fully recovered (self-report), number of newly diagnosed conditions (NDC), quality of life (EuroQoL-five dimension-five level (EQ-5D-5L) utility score compared with pre-COVID estimate) and healthcare resource costs (healthcare records). An analysis of the cost-effectiveness was performed by combining the healthcare resource cost and 1-year EQ-5D (giving a quality-adjusted life-year (QALY)) using statistical models that accounted for observed confounding.

**Results:**

At 1 year, 29% of participants felt fully recovered, and 41% of patients had an NDC. The most comprehensive services, where all patients could potentially access assessment, rehabilitation and mental health services, were more clinically effective when compared with either no service or light touch services (mean (SE) QALY 0.789 (0.012) vs 0.725 (0.026)), with an estimated cost per QALY of £1700 (95% uncertainty interval: dominated to £24 800).

**Conclusion:**

Our analysis supports the need for proactive, stratified, comprehensive follow-up, particularly assessment and rehabilitation for adults after hospitalisation with COVID-19, showing these services are likely to be both clinically and cost-effective according to commonly accepted thresholds.

**WHAT IS ALREADY KNOWN ON THIS TOPIC**

- At the time the COVID pandemic hit, little was known about the healthcare needs for patients who had been hospitalised. At the time, long covid had not been described as a phenomenon. Different regions of the UK were all providing care as best they could but using different models of care delivery.

**WHAT THIS STUDY ADDS**

- This study, commissioned by the UK National Institute for Health and Care Research and making use of the post-hospitalisation COVID-19 platform, sought to estimate the clinical effectiveness and cost-effectiveness of different healthcare pathways offered across the UK by looking at the first 12 months posthospital discharge. Using this unique data set and building on work previously described to categorise healthcare pathways, evidence on effectiveness and cost-effectiveness is generated. Overall, the evidence suggests that higher intensity of care following discharge for assessment and rehabilitation is associated with better outcomes at a cost that represents value for money for the health system.

**HOW THIS STUDY MIGHT AFFECT RESEARCH, PRACTICE OR POLICY**

- During the period of the study, additional clinical guidelines were developed that put in place recommendations for healthcare pathways to address the challenge of the emerging long covid/post-COVID condition. This study supports such interventions in showing the clinical and cost-effectiveness of comprehensive assessment and rehabilitation for these patients.

## Introduction

 Long covid remains a recognised ongoing health crisis. Despite the burden of disease, there is a limited evidence base to guide service models, diagnostic modalities and therapeutic interventions. Clinical care has evolved through expert opinion and experiential learning, with best practice advice and guidelines developed alongside.^1^,^3^ During the first year of the COVID-19 pandemic, healthcare pathways posthospitalisation for patients with severe COVID-19 were based on hospital teams making their own judgements about what follow-up they would provide and to which patients.^4^ In October 2020, in England, UK, a national long covid taskforce was formed, which included funding for specialist long covid clinics and a service specification was developed.^5^ To date, there is minimal published research on what long covid services were set up internationally.^6^ The evidence from this scoping review recommended that most long covid healthcare should be situated in primary care, and patients with complex symptoms should be referred to specialist long covid outpatient clinics, and depending on the patients’ needs, further referral to services such as rehabilitation should be considered.

Patients recovering from COVID-19 may experience new or worsening chronic conditions, for example, diabetes, cardiac disease, anxiety and depression, as well as ongoing symptoms in the absence of a defined chronic condition (long covid). As such, long covid can be a complex, multifactorial condition, and the UK National Institute for Health and Care Excellence (NICE) recommends the availability of integrated multidisciplinary rehabilitation services for complex cases.^2^ Emerging evidence in community observational studies suggests that long covid is associated with increased health service resource use^7^ and decreased quality of life.^8^ However, data on the effectiveness of rehabilitation in patients with long covid is limited.^9^ Most initial studies to date have been observational cohorts with no control group,^10^,^13^ which cannot account for natural recovery, while most randomised controlled trials are too small to be informative.^14^ The largest randomised controlled trial to date demonstrates the benefits of a remotely delivered supervised programme^9^ for patients posthospitalisation, and results are awaited for a face-to-face programme.^15^

We previously described and categorised healthcare pathways created for patients after discharge from hospital with COVID-19 at 45 hospital sites across the UK participating in the Post-HOSPitalisation COVID-19 (PHOSP-COVID) study at the time.^16 17^ This classification included whether there was a service available or not, and the level of complexity and/or comprehensiveness of service provided was assessed by four components: (1) which patients could access the service, for example, all patients versus only a subgroup such as only those who had received mechanical ventilation; (2) the level and complexity of the assessment; (3) the comprehensiveness of the rehabilitation service available and (4) the comprehensiveness of the mental health services on offer. For the assessment, comprehensiveness was determined by the availability of a face-to-face assessment, use of a multidisciplinary team, a multisystem approach and the availability of complex diagnostics. Higher comprehensiveness/complexity of the rehabilitation and mental health interventions included in the service required a multidimensional, holistic approach.

It is currently unclear how to optimally implement and stratify follow-up services to be holistic, integrated, equitable and both clinically and cost-effective. Understanding how to optimise healthcare support for individuals after severe COVID-19 to maximise quality of life and deliver services which are cost-effective is critical to personalised, high-quality, value for money, care. The latter was highlighted as a priority question by patients and clinicians.^18^ We therefore aimed to estimate the clinical and cost-effectiveness of identified pathways of posthospitalisation care available during the first year of the COVID-19 pandemic.

## Methods

### PHOSP-COVID dataset

We used data from the UK-based PHOSP-COVID cohort study.^19^ Participants were recruited from hospitals across the UK, having been discharged between February 2020 and March 2021 with a discharge diagnosis confirming, or a suspected illness caused by, COVID-19. Only participants from the sites where the health services survey was completed, so the healthcare pathway could be mapped, were used (34/45 tier 2 sites).^16^ Tier 2 refers to sites where patients attended for an in-person research visit (as opposed to tier 1, requiring consent to access patient healthcare records only). A variety of data were assessed, alongside detailed, holistic and multisystem assessments measured during participant follow-up visits, reported in detail elsewhere.^19^ The data included information relevant to the patient’s index admission, such as level of respiratory support, baseline health and demographics, health related quality of life (HRQoL) as measured by EuroQoL-five dimension (EQ-5D) instrument^20^; known comorbidities at hospital admission (including cardiac, respiratory, gastrointestinal, neurological and psychiatric, rheumatological, metabolic/endocrine/renal and malignancy/haematological) and information related to use of healthcare resources. Participants were also asked to retrospectively complete the EQ-5D-5L at the 5-month visit, estimating how they felt before their hospital admission for COVID-19 (pre-COVID).

In order to describe the post-COVID sequelae of this population, participants were asked whether they felt fully recovered from COVID-19 at around 5 and 12 months after discharge from hospital (available responses were yes, no or unsure) and newly diagnosed conditions (NDCs) were described. Indicators for NDCs were constructed from the data as conditions that were unrecorded prior to the hospital admission from COVID-19 and had a relevant objective investigation that was abnormal at 1 year postassessment: estimated glomerular filtration rate <60 mL/min/1.73 m^2^ in patients without a previous diagnosis of chronic kidney disease; glycated haemoglobin ≥6% in patients without a previous diagnosis of diabetes; Patient Health Questionnaire-9 ≥10 or Generalised Anxiety Disorder-7 >8 in patients without a previous diagnosis of depression or anxiety; Montreal Cognitive Assessment <23 in patients without a previous diagnosis of dementia; N-terminal pro-B-type natriuretic peptide ≥400 ng/L or B-type natriuretic peptide ≥100 ng/L in patients without a previous diagnosis of heart failure.

#### EQ-5D data and QALYs

We used the EQ-5D-5L version of the EQ-5D descriptive system to measure patient HRQoL.^21^ The survey assesses HRQoL across five dimensions: mobility, self-care, usual activities, pain/discomfort and anxiety/depression. Each dimension has five levels: no problems, slight problems, moderate problems, severe problems and extreme problems. Responses across the dimensions can be combined to give an overall utility index score, which summarises the patient’s HRQoL.

In line with UK NICE recommendations, we mapped EQ-5D-5L utility index scores to the three-level version of the score.^22^ The utility scores collected in PHOSP-COVID were employed to estimate the resulting quality-adjusted life-years (QALYs for the first year posthospital discharge based on the modelled analysis of EQ-5D outcomes.

#### Healthcare resource data and associated costs

To estimate patient healthcare resource use, we used self-report and available healthcare record data on primary, secondary and emergency care visits and medical investigations and procedures collected from bespoke clinical research forms at the two research visits. Unit cost data from the Health and Social Care Unit Cost database,^23^ the National Schedule of National Health Service costs^24^ and the Schedule of Events Cost Attribution Template^25^ were used to estimate the costs associated with healthcare resource use for the 2020 cost base year. Resource use items available in PHOSP-COVID and the derived unit costs used in the analysis are summarised in online supplemental appendix C table S1.

#### Healthcare pathways

**Figure 1:**
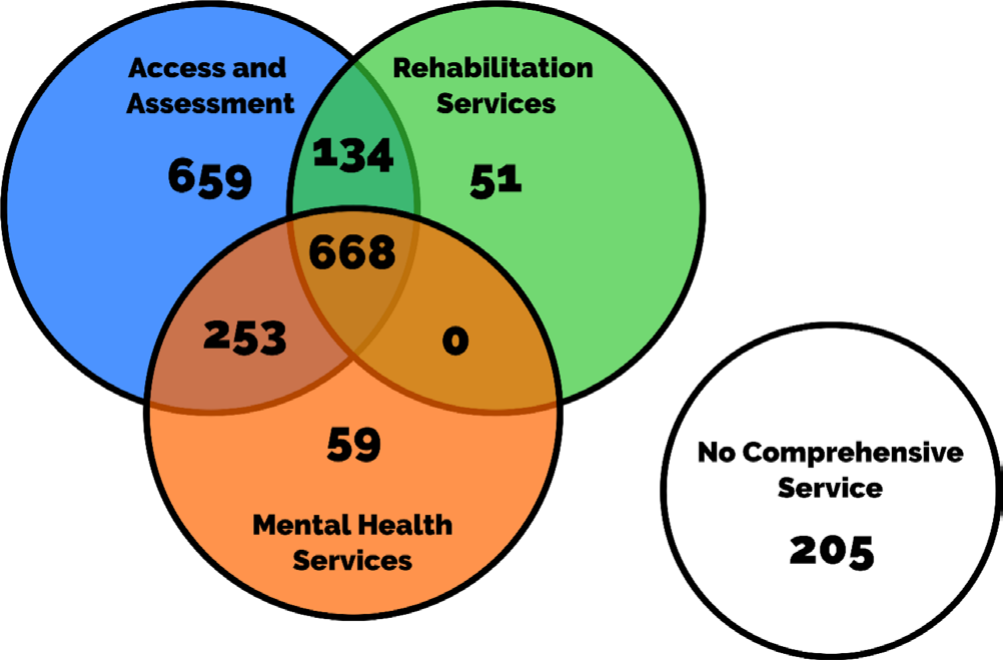
A Euler diagram to highlight patient numbers with access to comprehensive follow-up services for COVID-19 across the metrics of assessment, rehabilitation and mental health services. No comprehensive service indicates no comprehensive service for assessment, rehabilitation or mental health service and no follow-up service at all.

Based on the previously reported typology,^16^ we used four indicator variables: whether the comprehensiveness of assessment of posthospitalisation COVID services was low/high, whether the comprehensiveness of rehabilitation services was low/high, whether the comprehensiveness of mental health services was low/high and whether services were available for all patients or targeted only at a sub-group of patients (figure 1). Together, these four variables described 16 possible permutations of the healthcare pathway, of which 11 unique pathways were represented within the 45 sites of the PHOSP-COVID study. Those sites reporting ‘no service’ were considered to fall into the ‘low’ category of all four variables in the typology.

### Statistical analysis of PHOSP-COVID data

We aimed to adjust for observed case-mix variables in our estimation of the potential impacts of healthcare pathways on EQ-5D HRQoL/QALYs and health service resource costs. Available demographic, clinical and comorbidity data were used in a regression framework to estimate adjusted impacts of the four healthcare pathway variables described above. These were included in the regression equations as main effect variables, meaning that the 11 represented pathways in the PHOSP-COVID dataset were estimated by combining these four main effect variables estimated in the regression equations. Further detail of the precise form of these statistical models is presented in online supplemental appendix B of the supplementary materials. Alternative specifications of the models presented were explored, including using non-recovery and the existence of NDCs as mediating variables and as control variables (results not shown).

#### Approach to missing data and representativeness

In addition to the complete case analysis, we also undertook a two-step inverse probability weighting (IPW) analysis to reduce bias from missing data and to account for both the selection bias of the PHOSP-COVID cohort compared with the more representative International Severe Acute Respiratory and emerging Infection Consortium (ISARIC) study^26^ and for missing data; the precise methodology has been previously described.^27^ IPW can correct the potential bias and improve the representativeness compared with complete case analysis, although it is generally less efficient statistically than multiple imputation for handling missingness that is assumed to be missing at random.^28^ Nevertheless, it has the advantage that there is no need to create multiple complete data sets for analysis, and so it is more efficient at a practical level.

### Estimation of the cost-effectiveness of healthcare pathways

An analysis of the cost-effectiveness of the healthcare pathways seen in the PHOSP-COVID study was performed by combining the statistical equations for healthcare resource cost and 1-year EQ-5D (giving a QALY) for the different permutations of healthcare pathway offered, while holding all other variables in the regression constant at their mean values. Costs and QALYs for the different pathways identified are plotted on the cost-effectiveness plane with uncertainty represented by probabilistic sensitivity analysis.^29^

### Patient and public involvement (PPI) in the project

PPI representatives were involved in the project advisory group, which met every 3 months during the project. All members of the project advisory group were consulted and involved in the conduct of the project and helped with the dissemination of the project results. Two authors of the manuscript are patient representatives.

## Results

### Statistical analysis of PHOSP data

#### Descriptives

Out of 2697 tier 2 study participants, there were 2422 participants who were discharged from one of the 34 tier 2 sites that provided data, allowing their healthcare pathways to be mapped, and 2100 had a 1-year visit. Overall, 1013 participants were included in the analysis sample with complete data for all variables, including all the patient-reported outcome measures, the assessments for the NDCs and their summary demographic and baseline clinical information. There was good concordance in terms of baseline characteristics for the full sample and the analysis sample (table 1), but some differences remain. Most patients in the analysis sample were male (62%), white (79%), aged ≥50 years (81%) and had a body mass index of ≥30 kg/m^2^ (58%). The most common comorbidities at baseline were cardiac (46%), respiratory (28%) and neurological and/or psychiatric (19%). WHO class 5 (supplemental oxygen^30^) was the most common level of respiratory support provided during hospitalisation (42.3%). There were relatively equal numbers of patients across quintiles of social deprivation (18.1%–21.4%).

**Table 1:** Baseline demographic and clinical characteristics of analysis sample* and all those from sites with healthcare pathways mapped

Characteristic	Analysis sample (n=1013)		All available patients (n=2422)	
	n	(%)	n	(%)
Sex at birth				
Male	630	(62%)	1490	(62%)
Female	383	(38%)	931	(38%)
Missing			1	(0%)
Ethnicity				
White	795	(79%)	1815	(75%)
South Asian	87	(9%)	273	(11%)
Black	73	(7%)	170	(7%)
Mixed	22	(2%)	53	(2%)
Other	36	(4%)	111	(4%)
Missing			14	(1%)
WHO respiratory support class				
4	167	(17%)	407	(17%)
5	429	(42%)	1045	(43%)
6	219	(22%)	563	(23%)
7–9	198	(20%)	407	(17%)
Index of multiple deprivation quintile				
1 (most deprived)	216	(21%)	530	(22%)
2	217	(21%)	566	(23%)
3	188	(19%)	405	(17%)
4	183	(18%)	441	(18%)
5 (least deprived)	209	(21%)	469	(19%)
Missing			11	(0%)
Age at admission (years)				
<30	15	(2%)	56	(2%)
30–39	55	(5%)	146	(6%)
40–49	126	(12%)	371	(15%)
50–59	293	(29%)	700	(29%)
60–69	330	(33%)	694	(29%)
70–79	160	(16%)	375	(15%)
80+	34	(3%)	80	(3%)
Body mass index				
<30 kg/m^2^	423	(42%)	725	(30%)
≥30 kg/m^2^	590	(58%)	976	(40%)
Missing			721	(30%)
Presence of baseline comorbidity				
Cardiac	467	(46%)	1112	(46%)
Respiratory	281	(28%)	653	(27%)
Gastrointestinal	138	(14%)	330	(14%)
Neurological and psychiatric	196	(19%)	504	(21%)
Rheumatological	120	(12%)	130	(5%)
Metabolic/endocrine/renal	119	(12%)	294	(12%)
Malignancy/haematological	63	(6%)	263	(11%)
EuroQoL-five dimension prior to infection (recall)†	0.812	(0.22)	0.815	(0.231)
Missing			304	(13%)
Hospital site categorisation				
Assessment	898	(89%)	2107	(87%)
Rehabilitation services	439	(43%)	1246	(51%)
Mental health services	357	(35%)	980	(40%)
All patients offered service	591	(58%)	1577	(65%)

* Analysis sample is all those with complete data available.

† Continuous variable: mean (SD).

A summary of the healthcare pathway variables is provided in table 1. Most patients were discharged from a hospital site with comprehensive assessment services (89%), but there was less availability of comprehensive interventions, as only 43% of patients were discharged from a site offering a comprehensive rehabilitation service and 35% from a site offering a comprehensive mental health service. In total, 58% of patients were discharged from a site where follow-up services were available to all suitable patients, rather than restricted to a prespecified subgroup of patients. Patient numbers with access to comprehensive follow-up services for COVID-19 across the metrics of assessment, rehabilitation and mental health services based on a previously reported typology^16^ are shown in figure 1.

In total, 41% (415/1013) of patients had at least one NDC at 12 months that was not recorded at baseline hospital admission (table 2). The number and percentage of the included participants with an NDC of different types at 1 year posthospital admission are also shown in table 2.

**Table 2:** NDCs at 12 months from discharge

Chronic condition	Classification, N (%) 1013/100%
Chronic kidney disease	Estimated glomerular filtration rate <60 in patient without a previous diagnosis of chronic kidney disease, 94 (9.3%)
Diabetes	Glycated haemoglobin ≥6% in patient without a previous diagnosis of diabetes, 107 (10.6%)
Depression or anxiety	Patient Health Questionnaire-9≥10 or Generalised Anxiety Disorder-7 >8 in patient without previous diagnosis of depression or anxiety, 203 (20%)
Cognitive impairment	Montreal Cognitive Assessment <23 in patient without previous diagnosis of dementia, 79 (7.8%)
Cardiac dysfunction	pro-BNP ≥400 or BNP ≥100 in patient without previous diagnosis of heart failure 41 (4%)
Total	Any NDC 415 (41%)

BNP, B-type natriuretic peptide; NDC, newly diagnosed condition.

#### EQ-5D-3L utility scores

The median EQ-5D-3L utility score pre-COVID and at the first and second research visits was 0.889 (IQR 0.744–0.987), 0.753 (0.620–0.891) and 0.752 (0.581–0.893). The median difference in scores between the first research visit and pre-COVID was −0.072 (IQR −0.223 to 0.000), and between the second research visit and pre-COVID was −0.081 (IQR −0.232 to 0.000).

The EQ-5D utility score for participants who reported feeling fully recovered from their initial infection with COVID-19 and without an NDC was estimated to be 0.89 (online supplemental appendix C table S3), somewhat higher than would be expected based on national EQ-5D norm data.^31^ For subjects not feeling fully recovered but without an NDC, their utility was 0.13 units lower at 0.76. The lowest utility score, at 0.66, was for those individuals who were not feeling fully recovered and had an NDC. In general, unadjusted scores showed lower utility values for remaining symptoms and NDCs, reflecting the association between those health states and higher levels of comorbidity at baseline.

Results for the gamma-distributed log-link generalised linear model (GLM) for EQ-5D are presented in online supplemental appendix C table S3. Clinical and demographic characteristics associated with worse HRQoL were being female, receiving WHO class 7–9 (includes invasive mechanical ventilation) at hospitalisation compared with class 4 (no supplemental oxygen or other respiratory support)^30^, having a respiratory, neurological and/or psychiatric or a rheumatological comorbidity at baseline, and being obese. Conversely, characteristics associated with better HRQoL at 1 year were a higher pre-COVID utility index summary score and belonging to the least deprived index of multiple deprivation (IMD) quintile 5 compared with quintile 1.

**Figure 2:**
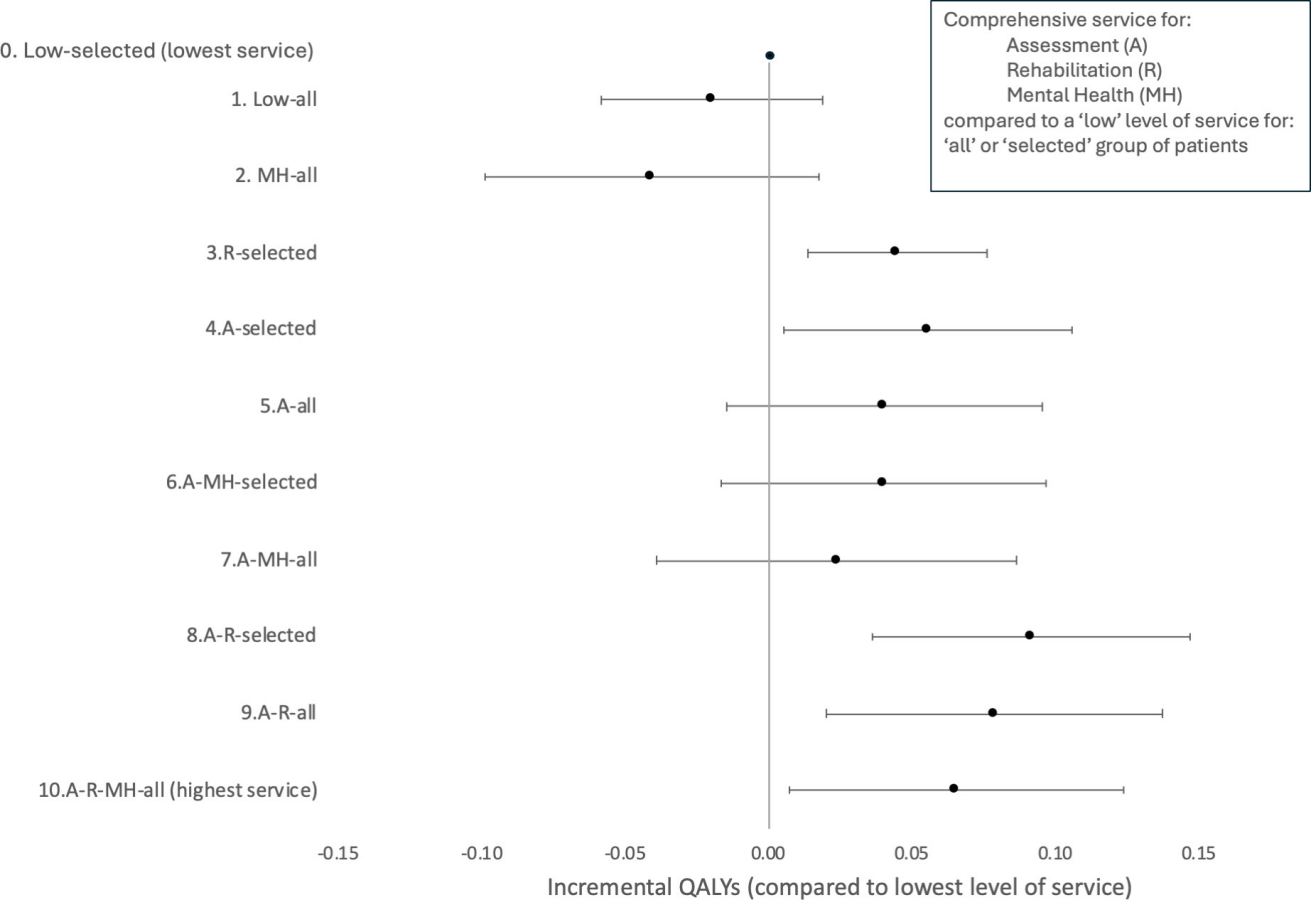
Forest plot of the impact on QALYs of each healthcare pathway represented in the Post-HOSPitalisation COVID-19 cohort compared with the lowest level of service available (lowest service included both no follow-up service and no comprehensive element of the service). ‘All’ refers to all patients who potentially could access the service, and ‘selected’ refers to only a prespecified subgroup that could access the service. QALYs, quality-adjusted life-years.

Controlling for all other covariates, access to comprehensive assessment and comprehensive rehabilitation services was both associated with significantly better HRQoL, which results in a significant estimate of quality-of-life benefit for four of the healthcare pathways estimated (figure 2).

#### Healthcare resource use and associated costs

The distribution of healthcare resource use costs was right-skewed, with a mean cost of just over £1000 per person and values ranging from around £0 to £55 000 per person. A gamma-distributed log-link GLM for healthcare cost at 12 months is presented in online supplemental appendix C table S4. Clinical and demographic characteristics that significantly increased healthcare cost were being female, receiving class 7–9 respiratory support at hospitalisation as opposed to class 4 and having a respiratory or malignancy/haematological comorbidity at baseline. Conversely, characteristics that significantly reduced healthcare costs were belonging to IMD quintiles 3–5 compared with the most deprived quintile (quintile 1) and being in age category 30–39 or category 60–69 compared with 50–59. Controlling for all other covariates, none of the healthcare pathway variables had a significant impact on healthcare costs at 12 months postdischarge. Online supplemental figure S1 in Appendix C shows the incremental health service resource costs estimated from the statistical models for each of 10 pathways compared with the lowest service pathway as a forest plot.

### Cost-effectiveness of healthcare pathways

**Figure 3:**
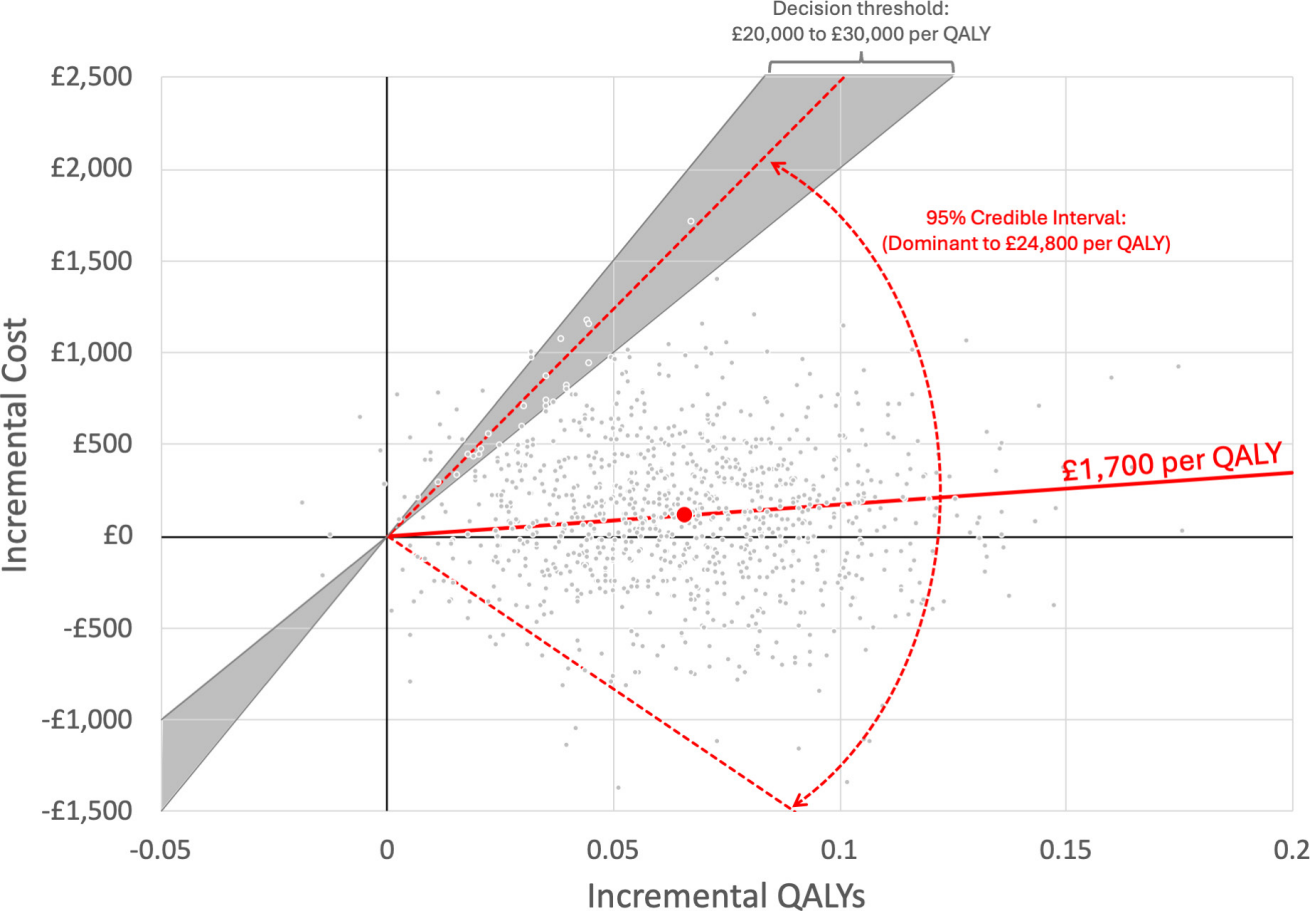
Comparison of the highest service pathway (10) to the lowest service pathway (0) on the cost-effectiveness plane. The red circle shows the point estimate of cost-effectiveness, the slope of the red solid line shows the incremental cost-effectiveness ratio, grey dots show uncertainty, and red dotted lines show the 95% credible interval for cost-effectiveness. QALYs, quality-adjusted life-years.

The estimated costs and effects based on the statistical models from online supplemental appendix C tables S3 and S4 are presented in online supplemental table S5 for each of the healthcare pathways represented in PHOSP-COVID. The highest healthcare pathway has an estimated incremental cost-effectiveness ratio of £1700 per QALY with CI in the dominant quadrant of the plane up to £24 800 per QALY (figure 3).

#### Sensitivity analysis weights

The regression models of online supplemental appendix C tables S2 and S3 were re-estimated using propensity score weights to adjust for potential missing at random effects of the missing data and to adjust the PHOSP cohort to look more representative of the true hospital population using ISARIC^26^ as a reference population. Online supplemental appendix C tables S6 and S7 show the reweighted regression analyses. Overall, coefficients from the regression models were not substantially different when using the weighted analyses; consequently, neither was the estimated cost-effectiveness of the highest level of service (which reduced slightly to under £1000 per QALY). Alternative specifications of the models, including using non-recovery and the existence of NDCs as mediating variables and as control variables, did not change the results markedly (results not shown).

## Discussion

We report for the first time that healthcare pathways for adult survivors of a hospital admission for COVID-19 appear to be clinically effective if they offer a comprehensive service. The most comprehensive services, where all patients could potentially access comprehensive assessment, rehabilitation and mental health services, were clinically effective when compared with either no service or ‘light touch’ (lowest) services (figure 2). The most comprehensive service was also cost-effective compared with no service or ’light touch’ services (lowest) (figure 3) with an estimated cost per QALY of £1700 (95% uncertainty interval: dominated to £24 800).

Our results particularly support the effectiveness of a comprehensive assessment and the availability of multidimensional rehabilitation. To our knowledge, this is the first description highlighting the clinical effectiveness of a complex/comprehensive assessment. The comprehensive assessment included a face-to-face option, a multisystem approach, complex diagnostics and the availability of a multidisciplinary and interspeciality team meeting. To date, the type of follow-up assessment provided (for any of the healthcare pathways) has been based on expert opinion. The NHS England long covid service specification is one of the very few internationally designed and implemented at a national level.^32^ Our data support the components described in the service specification.

Our data suggest that multidimensional rehabilitation is clinically effective, which supports systematic reviews of small-scale randomised controlled trials investigating rehabilitation versus usual care that suggest effectiveness,^14^ although definitive trials are needed. The largest randomised controlled trial to date reported a difference in the EQ-5D-5L uncertainty interval of 0.02 (−0.01 to 0.05) units with the digital rehabilitation intervention compared with usual care at 3 months. Our data suggests a larger difference with rehabilitation interventions than the Rehabilitation Exercise and psycholoGical support After covid-19 InfectioN (REGAIN) trial, which might be due to the face-to-face intervention offered at some sites.^9 16^

Our data are perhaps less certain for mental health interventions in isolation, and most exercise-based rehabilitation programmes also contain interventions to support mental health, and some are integrated, which we may not have captured from the survey. A systematic review of registered trials for interventions for mental health, cognition and psychological well-being in long covid highlighted that the breadth and scope of research remains limited. Our data highlight a significant new burden of symptoms suggestive of anxiety and depression (the challenges of interpretation of the questionnaires in a physically unwell population notwithstanding) and therefore highlight the urgent need for interventions to improve both physical and mental health. The categorisation of services offered to all patients or a select group of patients did not seem to have a large impact on the results in our cohort study, but we would recommend that all patients with potential need to have access to services rather than a prespecified criterion.

Although our data show clinical effectiveness for the more comprehensive services, there is a balance between the cost of a comprehensive service for all patients to access versus either limiting it to those with the most severe acute disease or only providing a light-touch service, such as a one-off telephone call or no service at all. We report positive data on cost per QALY, suggesting that the most comprehensive service is both clinical and cost-effective based on commonly accepted thresholds for cost-effectiveness in the £20 000–30 000 per QALY range.^33^

In all, only 29% of patients report feeling fully recovered from COVID at 1 year after discharge. NDCs were apparent in 46% of participants, which could account for the remaining symptoms, leaving 39% reporting sustained symptoms at 1 year with no clear cause. This is the closest group in our data to the definition of post-COVID-19 condition (long covid) by the WHO.^34^ However, it is an underestimate of the prevalence of long covid, as it assumes that, in patients with an NCD, these conditions fully account for their persistent symptoms, which is unlikely. In addition to our previous reports of low rates of patient-perceived recovery at 1 year after discharge from hospital in the PHOSP-COVID study,^35^ we highlight a large new health burden of NDCs such as diabetes, new mental health symptoms and cognitive impairment. While we concede that some of these could have been pre-existing before COVID-19 but undiagnosed, many will be as a result of (or exacerbated by) COVID-19. These long-term consequences of COVID-19 require optimised treatment, which supports the need for multispeciality expertise being available for long covid clinics.^2^

### Strengths and limitations

The strengths of the data are the detailed objective follow-up of a large number of participants alongside the detailed characterisation of the long covid follow-up at their hospital site. Selection and survivor bias (the cohort are survivors to 1 year after discharge) were mitigated by modelling to both the larger PHOSP-COVID cohort and to the ISARIC data set (a larger cohort of patients admitted into a UK hospital for COVID-19).

However, there are important limitations to be considered. Although attempts have been made to control for observed confounding, given that this is an observational study, it is likely that unobserved confounding remains. Furthermore, the process of controlling for observed confounders meant that only a subset of the overall data was used. Despite statistical adjustment for missing data, the full consequences of that missing data add to the uncertainty over the study’s results. There was difficulty in determining the precise level of post-COVID-19 services on offer for individual participants, as this information was mapped at the site level from survey data and is therefore not a direct assessment of services. Although the services did not alter significantly over the first two waves of the pandemic in the UK,^16^ there may have been some changes in the second year not accounted for in our analysis, which also estimates only the main effects of the service (unreported analyses revealed no significant interaction terms, but their existence cannot be ruled out). These main effects were included without regard to their statistical significance, although the uncertainty in the estimation was captured in the model estimation. Since the statistical models estimated main effects on the relative scale, the estimates of the absolute effect will depend, to some extent, on the baseline values of the hospitalised patients. Alternative specifications of the models were tested but did not materially impact the reported results in this manuscript.

The PHOSP study participants were discharged from the hospital between February 2020 and 31 March 2021 and were therefore mostly unvaccinated prior to hospital admission and before use of most therapeutics for acute COVID-19. Therefore, our data represents what services worked well at the start of the pandemic, which can be used for future pandemics. Due to higher vaccination rates, better acute treatments for COVID-19 and new variants of the disease, it is unknown if our data remains applicable for contemporary patients who are nevertheless serious enough to be hospitalised for their acute infection, but it is likely. Furthermore, some groups of patients, such as the immunocompromised population, have remained at the same high risk of severe disease through the pandemic despite vaccination.^36^ Our data is for patients with severe COVID-19 and cannot be directly extrapolated to non-hospitalised cases of long covid. However, the comprehensive clinical care model is applicable as described by the NHS England service specification. Clinical and cost-effectiveness require further evaluation in the non-hospitalised population. The hospital admission data were not retrieved from the NHS linkage but were retrieved by researchers from the patients’ medical records. For example, an admission at a different location may not have been known about if the participant did not recall it.

### Clinical implications

To date, long covid care is heterogeneous across the UK and internationally. Our data support the need for proactive care and for a clinically and cost-effective comprehensive care model for assessment, rehabilitation and mental health services. This is predominantly to improve health-related quality of life for individuals, which is similarly reduced in our data compared with other long-term conditions.^37^ However, there are additional benefits to dedicated long covid clinics, such as developing teams of healthcare professionals that are experts in this complex multisystem disease and who could collectively run clinical trials of much-needed treatments in eligible patients. Other benefits include establishing correct coding of health records and helping the industry understand the healthcare models their products would be prescribed within if clinical trials were successful.

### Summary

In summary, comprehensive healthcare models for assessment and rehabilitation for adult survivors of a hospital admission for COVID-19 are estimated to be clinically effective and cost-effective compared with commonly accepted thresholds. Further work needs to be extended to healthcare models for the larger group of non-hospitalised patients who develop long covid.

## Supplementary material

10.1136/bmjresp-2025-003224 online supplemental file 1

## Data Availability

Data are available upon reasonable request.
