# Supplementary material for: Clinical and cost-effectiveness of diverse posthospitalisation pathways for COVID-19: a UK evaluation using the PHOSP-COVID cohort
Source: BMJ Open Respir Res. 2025 Oct 31;12(1):e003224. doi: 10.1136/bmjresp-2025-003224 (PMC12581062; doi:10.1136/bmjresp-2025-003224)
Supplement: online supplemental file 1 [file bmjresp-12-1-s001.docx]

SUPPLEMENTARY MATERIALS

**Appendix A: PHOSP-COVID Collaborative Group**

# PHOSP-COVID Collaborative Group

**Core Management Group**

*Chief Investigator* C E Brightling, *Members* R A Evans (Lead Co-I), L V Wain (Lead Co-I), J D Chalmers, V C Harris, L P Ho, A Horsley, M Marks, K Poinasamy, B Raman, A Shikotra, A Singapuri

**PHOSP-COVID Study Central Coordinating Team**

C E Brightling (Chief Investigator), R A Evans (*Lead Co-I*), L V Wain (*Lead Co-I*), R Dowling, C Edwardson, O Elneima, S Finney, N J Greening, B Hargadon, V C Harris, L Houchen--Wolloff, O C Leavy, H J C McAuley, C Overton, T Plekhanova, R M Saunders, M Sereno, A Singapuri, A Shikotra, C Taylor, S Terry, C Tong, B Zhao

**Steering Committee**

*Co-chairs* D Lomas, E Sapey*, Institution representatives* C Berry, C E Bolton, N Brunskill, E R Chilvers, R Djukanovic, Y Ellis, D Forton, N French, J George, N A Hanley, N Hart, L McGarvey, N Maskell, H McShane, M Parkes, D Peckham, P Pfeffer, A Sayer, A Sheikh, A A R Thompson, N Williams and core management group representation

**Executive Board**

*Chair* C E Brightling, representation from the core management group, each working group and platforms

**Platforms**

**Bioresource**

W Greenhalf (*Co-Lead*), M G Semple (*Co-Lead*), M Ashworth, H E Hardwick, L Lavelle-Langham, W Reynolds, M Sereno, R M Saunders, A Singapuri, V Shaw, A Shikotra, B Venson, L V Wain

**Data Hub**

A B Docherty (*Co-Lead*), E M Harrison (*Co-Lead*), A Sheikh (*Co-Lead*), J K Baillie, C E Brightling, L Daines, R Free, R A Evans, S Kerr, O C Leavy, N I Lone, D Lozano-Rojas, H J C McAuley, K Ntotsis, R Pius, J Quint, M Richardson, , M Sereno, M Thorpe, L V Wain

**Genetic Analysis**

L V Wain (Co-Lead), J K Baillie (*Co-Lead*), N Avramidis, E Coughlan, B Guillen-Guio, O C Leavy, E Pairo-Castineira, K Rawlik

**Imaging Alliance**

M Halling-Brown (*Co-Lead*), F Gleeson (*Co-Lead*), J Jacob (*Co-Lead*), S Neubauer (*Co-Lead*) B Raman (*Co-Lead*) S Siddiqui (*Co-Lead*) J M Wild (*Co-Lead*), S Aslani, G Baxter, M Beggs, C Bloomfield, M P Cassar, A Chiribiri, E Cox, D J Cuthbertson, M Halling-Brown, V M Ferreira, L Finnigan, S Francis, P Jezzard, G J Kemp, H Lamlum, E Lukaschuk, C Manisty, G P McCann , C McCracken, K McGlynn , R Menke , C A Miller , A J Moss, T E Nichols, C Nikolaidou , C O’Brien , G Ogbole, B Rangelov, D P O’Regan , A Pakzad, S Piechnik , S Plein, I Propescu, A A Samat, L Saunders, Z B Sanders, R Steeds, T Treibel, E M Tunnicliffe, M Webster, J Willoughby, J Weir McCall, C Xie, M Xu

**Omics**

L V Wain (*Co-Lead)*, J K Baillie (*Co-Lead*), H Baxendale, C E Brightling, M Brown, J D Chalmers, R A Evans, B Gooptu, W Greenhalf, H E Hardwick, R G Jenkins, D Jones, I Koychev, C Langenberg, A Lawrie, P L Molyneaux, A Shikotra, J Pearl, M Ralser, N Sattar, R M Saunders, J T Scott, T Shaw, D Thomas, D Wilkinson

**Working Groups**

**Airways**

L G Heaney (*Co-Lead*), A De Soyza (*Co-Lead*), D Adeloye, C E Brightling, J S Brown, J Busby, J D Chalmers, C Echevarria, L Daines, O Elneima, RA Evans, J Hurst, P Novotny, C Nicolaou, P Pfeffer, K Poinasamy, J Quint, I Rudan, E Sapey, M Shankar-Hari, A Sheikh, S Siddiqui, S Walker, B Zheng

**Brain**

J R Geddes (*Lead*), M Hotopf *(Co-Lead),* K Abel, R Ahmed, L Allan, C Armour, D Baguley, D Baldwin, C Ballard, K Bhui, G Breen, K Breeze, M Broome, T Brugha, E Bullmore, D Burn, F Callard, J Cavanagh, T Chalder, D Clark, A David, B Deakin, H Dobson, B Elliott, J Evans, RA Evans, R Francis, E Guthrie, P Harrison, M Henderson,  A Hosseini, N Huneke, M Husain, T Jackson, I Jones, T Kabir, P Kitterick, A Korszun, I Koychev, J Kwan, A Lingford-Hughes, P Mansoori, H McAllister-Williams, K McIvor, B Michael, L Milligan, R Morriss, E Mukaetova-Ladinska, K Munro, A Nevado-Holgado, T Nicholson, C Nicolaou, S Paddick, C Pariante, J Pimm, K Saunders, M Sharpe, G Simons, J P Taylor, R Upthegrove, S Wessely

**Cardiac**

G P McCann (*Lead*), S Amoils, C Antoniades, A Banerjee, A Bularga, C Berry, P Chowienczyk, J P Greenwood, A D Hughes, K Khunti, C Lawson, N L Mills, A J Moss, S Neubauer, B Raman, A N Sattar, C L Sudlow, M Toshner,

**Immunology**

P J M Openshaw (*Lead*), D Altmann, J K Baillie, R Batterham, H Baxendale, N Bishop, C E Brightling, P C Calder, C M Efstathiou, R A Evans, J L Heeney, T Hussell, P Klenerman, F Liew, J M Lord, P Moss, S L Rowland-Jones, W Schwaeble, M G Semple, R S Thwaites, L Turtle, L V Wain, S Walmsley, D Wraith

**Intensive Care**

M J Rowland (*Lead*), A Rostron (*Co-Lead*), J K Baillie, B Connolly, A B Docherty, N I Lone, D F McAuley, D Parekh, A Rostron, J Simpson, C Summers

**Lung Fibrosis**

R G Jenkins (*Co-Lead*), J Porter (*Co-Lead*), R J Allen, R Aul, J K Baillie, S Barratt, P Beirne, J Blaikley, R C Chambers, N Chaudhuri, C Coleman, E Denneny, L Fabbri, P M George, M Gibbons, F Gleeson, B Gooptu, B Guillen-Guio, I Hall, N A Hanley, L P Ho, E Hufton, J Jacob, I Jarrold, G Jenkins, S Johnson, M G Jones, S Jones, F Khan, P Mehta, J Mitchell, P L Molyneaux, J E Pearl, K Piper Hanley, K Poinasamy, J Quint, D Parekh, P Rivera-Ortega, L C Saunders, M G Semple, J Simpson, D Smith, M Spears, L G Spencer, S Stanel, I Stewart, A A R Thompson, D Thickett, R Thwaites, L V Wain, S Walker, S Walsh, J M Wild, D G Wootton, L Wright

**Metabolic**

S Heller (*Co-Lead*), M J Davies (*Co-Lead*), H Atkins, S Bain, J Dennis, K Ismail, D Johnston, P Kar, K Khunti, C Langenberg, P McArdle, A McGovern, T Peto, J Petrie, E Robertson, N Sattar, K Shah, J Valabhji, B Young

**Pulmonary and Systematic Vasculature**

L S Howard (*Co-Lead*), Mark Toshner (*Co-Lead*), C Berry, P Chowienczyk, A Lawrie, O C Leavy, J Mitchell, J Newman, L Price, J Quint, A Reddy, J Rossdale, N Sattar, C Sudlow, A A R Thompson, J M Wild, M Wilkins

**Rehabilitation, Sarcopenia and Fatigue**

S J Singh (*Co-Lead*), W D-C Man (*Co-Lead*), J M Lord (*Co-Lead*), N J Greening (*Co-Lead*), T Chalder (*Co-Lead*), J T Scott (*Co-Lead*), N Armstrong, E Baldry, M Baldwin, N Basu, M Beadsworth, L Bishop, C E Bolton, A Briggs, M Buch, G Carson, J Cavanagh, H Chinoy, C Dawson, E Daynes, S Defres, R A Evans, L Gardiner, P Greenhaff, S Greenwood, M Harvie, L HOuchen-Wolloff, M Husain, S MacDonald, A McArdle, H J C McAuley, A McMahon, M McNarry, G Mills, C Nolan, K O’Donnell, D Parekh, Pimm, J Sargent, L Sigfrid, M Steiner, D Stensel, A L Tan, I Vogiatzis, J Whitney, D Wilkinson, D Wilson, M Witham, D G Wootton, T Yates

**Renal**

D Thomas (*Lead*), N Brunskill (*Co-Lead*), S Francis (*Co-Lead*), S Greenwood (*Co-Lead*), C Laing (*Co-Lead*), K Bramham, P Chowdhury, A Frankel, L Lightstone, S McAdoo, K McCafferty, M Ostermann, N Selby, C Sharpe, M Willicombe

**Patient Public Engagement Group**

L Houchen-Wolloff (*Lead*), J Bunker, R Gill, C Hastie, R Nathu, N Rogers, N Smith

**Local Clinical Centre PHOSP-COVID trial staff**

(listed in alphabetical order)

**Airedale NHS Foundation Trust**

A Shaw (PI), L Armstrong, B Hairsine, H Henson, C Kurasz, L Shenton

**Aneurin Bevan University Health Board**

S Fairbairn (PI), A Dell, N Hawkings, J Haworth, M Hoare, A Lucey, V Lewis, G Mallison, H Nassa, C Pennington, A Price, C Price, A Storrie, G Willis, S Young

**Barts Health NHS Trust &** **Queen Mary University of London**

P Pfeffer (PI), K Chong-James, C David, W Y James, C Manisty, A Martineau, O Zongo

**Barnsley Hospital NHS Foundation Trust**

A Sanderson (PI)

**Belfast Health and Social Care Trust & Queen's University Belfast**

L G Heaney (PI), C Armour, V Brown, T Craig, S Drain, B King, N Magee, D McAulay, E Major, L McGarvey, J McGinness, R Stone

**Betsi Cadwaladr University Health Board**

A Haggar (PI), A Bolger, F Davies, J Lewis, A Lloyd, R Manley, E McIvor, D Menzies, K Roberts, W Saxon, D Southern, C Subbe, V Whitehead

**Borders General Hospital, NHS Borders**

H El-Taweel (PI), J Dawson, L Robinson

**Bradford Teaching Hospitals NHS Foundation Trust**

D Saralaya (PI), L Brear, K Regan, K Storton

**Cambridge University Hospitals NHS Foundation Trust, NIHR Cambridge Clinical Research Facility & University of Cambridge**

J Fuld (PI), A Bermperi, I Cruz, K Dempsey, A Elmer, H Jones, S Jose, S Marciniak, M Parkes, C Ribeiro, J Taylor, M Toshner, L Watson, J Weir McCall, J Worsley

**Cardiff and Vale University Health Board**

R Sabit (PI), L Broad, A Buttress, T Evans, M Haynes, L Jones, L Knibbs, A McQueen, C Oliver, K Paradowski, J Williams

**Chesterfield Royal Hospital NHS Trust**

E Harris (PI), C Sampson

**Cwm Taf Morgannwg University Health Board**

C Lynch (PI), E Davies, C Evenden , A Hancock, K Hancock, M Rees , L Roche, N Stroud, T Thomas-Woods

**East Cheshire NHS Trust**

M Babores (PI), J Bradley-Potts, M Holland, N Keenan, S Shashaa, H Wassall

**East Kent Hospitals University NHS Foundation Trust**

E Beranova (PI), H Weston (PI), T Cosier, L Austin, J Deery, T Hazelton, C Price, H Ramos, R Solly, S Turney

**Gateshead NHS Trust**

L Pearce (PI), W McCormick, S Pugmire, W Stoker, A Wilson

**Guy’s and St Thomas’ NHS Foundation Trust**

N Hart (PI), LA Aguilar Jimenez, G Arbane, S Betts, K Bisnauthsing, A Dewar, P Chowdhury, A Chiribiri, A Dewar, G Kaltsakas, H Kerslake, MM Magtoto, P Marino, LM Martinez, C O'Brien, M Ostermann, J Rossdale, TS Solano, E Wynn

**Hampshire Hospitals NHS Foundation Trust**

N Williams (PI), W Storrar (PI), M Alvarez Corral, A Arias, E Bevan, D Griffin, J Martin, J Owen,

S Payne, A Prabhu, A Reed, C Wrey Brown

**Harrogate and District NHD Foundation Trust**

C Lawson (PI), T Burdett, J Featherstone, A Layton, C Mills, L Stephenson,

**Hull University Teaching Hospitals NHS Trust & University of Hull**

N Easom (PI), P Atkin, K Brindle, M G Crooks, K Drury, R Flockton, L Holdsworth, A Richards, D L Sykes, S Thackray-Nocera, C Wright

**Hywel Dda University Health Board**

K E Lewis (PI), A Mohamed (PI), G Ross (PI), S Coetzee, K Davies, R Hughes, R Loosley, L O’Brien, Z Omar, H McGuinness, E Perkins, J Phipps, A Taylor, H Tench, R Wolf-Roberts

**Imperial College Healthcare NHS Trust & Imperial College London**

L S Howard (PI), O Kon (PI), D C Thomas (PI), S Anifowose, L Burden, E Calvelo, B Card, C Carr, E R Chilvers, D Copeland, P Cullinan, P Daly, C M Efstathiou, L Evison, T Fayzan, H Gordon, S Haq, R G Jenkins, C King, F Liew, K March, M Mariveles, L McLeavey, N Mohamed, S Moriera, U Munawar, J Nunag, U Nwanguma, L Orriss- Dib, D P O'Regan, A Ross, M Roy, E Russell, K Samuel, J Schronce, N Simpson, L Tarusan, C Wood, N Yasmin

**Kettering General Hospital NHS Trust**

R Reddy (PI), A-M, Guerdette, M Hewitt, K Warwick, S White

**King’s College Hospital NHS Foundation Trust & Kings College London**

A M Shah (PI), C J Jolley (PI), O Adeyemi, R Adrego, H Assefa-Kebede, J Breeze, M Brown, S Byrne, T Chalder, A Chiribiri, P Dulawan, N Hart, A Hayday, A Hoare, A Knighton, M Malim, C O'Brien, S Patale, I Peralta, N Powell, A Ramos, K Shevket, F Speranza, A Te

**Leeds Teaching Hospitals & University of Leeds**

P Beirne (PI), A Ashworth, J Clarke, C Coupland, M Dalton, E Wade, C Favager, J Greenwood, J Glossop, L Hall, T Hardy, A Humphries, J Murira, D Peckham, S Plein, J Rangeley, G Saalmink, A L Tan, B Whittam, N Window, J Woods,

**Lewisham & Greenwich NHS Trust**

G Coakley (PI)

**Liverpool University Hospitals NHS Foundation Trust & University of Liverpool**

D G Wootton (PI), L Turtle (PI), L Allerton, AM All, M Beadsworth, A Berridge, J Brown, S Cooper, A Cross, D J Cuthbertson, S Defres, S L Dobson, J Earley, N French, W Greenhalf, H E Hardwick, K Hainey, J Hawkes, V Highett, S Kaprowska, G J Kemp, AL Key, S Koprowska, L Lavelle-Langham, N Lewis-Burke, G Madzamba, F Malein, S Marsh, C Mears, L Melling, M J Noonan, L Poll, J Pratt, E Richardson, A Rowe, M G Semple, V Shaw, K A Tripp, B Vinson, L O Wajero, S A Williams-Howard, J Wyles

**London North West University Healthcare NHS Trust**

S N Diwanji (PI), P Papineni (PI), S Gurram, S Quaid, G F Tiongson, E Watson

**Manchester University NHS Foundation Trust & University of Manchester**

B Al-Sheklly (PI), A Horsley (PI), C Avram, P Barran, J Blaikely, M Buch, N Choudhury, D Faluyi, T Felton, T Gorsuch, N A Hanley, T Hussell, Z Kausar, C A Miller, N Odell, R Osbourne, K Piper Hanley, K Radhakrishnan, S Stockdale, D Trivedi

**Newcastle upon Tyne Hospitals NHS Foundation Trust & University of Newcastle**

A De Soyza (PI), C Echevarria (PI), A Ayoub, J Brown, G Burns, G Davies, H Fisher, C Francis, A Greenhalgh, P Hogarth, J Hughes, K Jiwa, G Jones, G MacGowan, D Price, A Sayer, J Simpson, H Tedd, S Thomas, S West, M Witham, S Wright, A Young

**NHS Dumfries and Galloway**

M J McMahon (PI), P Neill

**NHS Greater Glasgow and Clyde Health Board & University of Glasgow**

D Anderson (PI), H Bayes (PI), C Berry (PI), D Grieve (PI), I B McInnes (PI), N Basu, A Brown, A Dougherty, K Fallon, L Gilmour, K Mangion, A Morrow, K Scott, R Sykes, R Touyz

**NHS Highland**

E K Sage (PI), F Barrett, A Donaldson

**NHS Lanarkshire**

M Patel (PI), D Bell, A Brown, M Brown, R Hamil, K Leitch, L Macliver, J Quigley, A Smith, B Welsh

**NHS Lothian & University of Edinburgh**

G Choudhury (PI), J K Baillie, S Clohisey, A Deans, A B Docherty, J Furniss, E M Harrison, S Kelly, N I Lone, D E Newby, A Sheikh

**NHS Tayside & University of Dundee**

J D Chalmers (PI), D Connell, A Elliott, C Deas, J George, S Mohammed, J Rowland, A R Solstice, D Sutherland, C J Tee

**North Bristol NHS Trust & University of Bristol**

N Maskell (PI), D Arnold, S Barrett, H Adamali, A Dipper, S Dunn, A Morley, L Morrison, L Stadon, S Waterson, H Welch

**North Middlesex Hospital NHS Trust**

B Jayaraman (PI), T Light

**Nottingham University Hospitals NHS Trust & University of Nottingham**

C E Bolton (PI), P Almeida, J Bonnington, M Chrystal, E Cox, C Dupont, S Francis, P Greenhaff, A Gupta, L Howard, W Jang, S Linford, L Matthews, R Needham, A Nikolaidis, S Prosper, K Shaw, A K Thomas

**Oxford University Hospitals NHS Foundation Trust & University of Oxford**

L P Ho (PI), N M Rahman (PI), M Ainsworth, A Alamoudi, M Beggs, A Bates, A Bloss, A Burns, P Carter, M Cassar, K M Channon, J Chen, F Conneh, T Dong, R I Evans, E Fraser, X Fu, J R Geddes, F Gleeson, P Harrison, M Havinden-Williams, P Jezzard, N Kanellakis, I Koychev, P Kurupati, X Li, E Lukaschuk, K McGlynn, H McShane, C Megson, K Motohashi, S Neubauer, D Nicoll, G Ogg, E Pacpaco, M Pavlides, Y Peng, N Petousi, J Propescu, N Rahman, B Raman, M J Rowland, K Saunders, M Sharpe, N Talbot, E Tunnicliffe

**Royal Brompton and Harefield Clinical Group, Guy’s and St Thomas’ NHS Foundation Trust.**

W D-C Man (PI), B Patel (PI), R E Barker, D Cristiano, N Dormand, M Gummadi, S Kon, K Liyanage, C M Nolan, S Patel, O Polgar, P Shah, S J Singh, J A Walsh

**Royal Free London NHS Foundation Trust**

J Hurst (PI), H Jarvis (PI), S Mandal (PI), S Ahmad, S Brill, L Lim, D Matila, O Olaosebikan, C Singh

**Royal Papworth Hospital NHS Foundation Trust**

M Toshner (PI), H Baxendale, L Garner, C Johnson, J Mackie, A Michael, J Pack, K Paques, H Parfrey, J Parmar

**Salford Royal NHS Foundation Trust**

N Diar Bakerly (PI), P Dark, D Evans, E Hardy, A Harvey, D Holgate, S Knight, N Mairs, N Majeed, L McMorrow, J Oxton, J Pendlebury, C Summersgill, R Ugwuoke, S Whittaker

**Salisbury NHS Foundation Trust**

W Matimba-Mupaya (PI), S Strong-Sheldrake

**Sheffield Teaching NHS Foundation Trust & University of Sheffield**

S L Rowland-Jones (PI), A A R Thompson (Co PI), J Bagshaw, M Begum, K Birchall, R Butcher, H Carborn, F Chan, K Chapman, Y Cheng, L Chetham, C Clark, Z Coburn, J Cole, M Dixon, A Fairman, J Finnigan, L Finnigan, H Foot, D Foote, A Ford, R Gregory, K Harrington, L Haslam, L Hesselden, J Hockridge, A Holbourn, B Holroyd-Hind, L Holt, A Howell, E Hurditch, F Ilyas, C Jarman, A Lawrie, E Lee, J-H Lee, R Lenagh, A Lye, I Macharia, M Marshall, A Mbuyisa, J McNeill, S Megson, J Meiring, L Milner, S Misra, H Newell, T Newman, C Norman, L Nwafor, D Pattenadk, M Plowright, J Porter, P Ravencroft, C Roddis, J Rodger, P Saunders, J Sidebottom, J Smith, L Smith, N Steele, G Stephens, R Stimpson, B Thamu, N Tinker, K Turner, H Turton, P Wade, S Walker, J Watson, J M Wild, I Wilson, A Zawia

**St George’s University Hospitals NHS Foundation Trust**

R Aul (PI), M Ali, A Dunleavy (PI), D Forton, N Msimanga, M Mencias, T Samakomva, S Siddique, J Teixeira, V Tavoukjian

**Sherwood Forest Hospitals NHS Foundation Trust**

J Hutchinson (PI), L Allsop, K Bennett, P Buckley, M Flynn, M Gill, C Goodwin, M Greatorex, H Gregory, C Heeley, L Holloway, M Holmes, J Kirk, W Lovegrove, TA Sewell, S Shelton, D Sissons, K Slack, S Smith, D Sowter, S Turner, V Whitworth, I Wynter

**Shropshire Community Health NHS Trust**

L Warburton (PI), S Painter, J Tomlinson

**Somerset NHS Foundation Trust**

C Vickers (PI), T Wainwright, D Redwood, J Tilley, S Palmer

**Swansea Bay University Health Board**

G A Davies (PI), L Connor, A Cook, T Rees, F Thaivalappil, C Thomas

**Tameside and Glossop Integrated Care NHS Foundation**

A Butt (PI), M Coulding, H Jones, S Kilroy, J McCormick, J McIntosh, H Savill, V Turner, J Vere

**The Great Western Hospital Foundation Trust**

E Fraile (PI), J Ugoji

**The Hillingdon Hospitals NHS Foundation Trust**

S S Kon (PI), H Lota, G Landers, M Nasseri, S Portukhay

**The Rotherham NHS Foundation Trust**

A Hormis (PI), A Daniels, J Ingham, L Zeidan

**United Lincolnshire Hospitals NHS Trust**

M Chablani (PI), L Osborne

**University College London Hospital & University College London**

M Marks (PI), J S Brown (PI), N Ahwireng, B Bang, D Basire, R C Chambers, A Checkley, R Evans, M Heightman, T Hillman, J Hurst, J Jacob, S Janes, R Jastrub, M Lipman, S Logan, D Lomas, M Merida Morillas, A Pakzad, H Plant, J C Porter, K Roy, E Wall, B Williams, M Xu

**University Hospital Birmingham NHS Foundation Trust & University of Birmingham**

D Parekh (PI), N Ahmad Haider, C Atkin, R Baggott, M Bates, A Botkai, A Casey, B Cooper, J Dasgin, K Draxlbauer, N Gautam, J Hazeldine, T Hiwot, S Holden, K Isaacs, T Jackson, S Johnson, V Kamwa, D Lewis,

J M Lord, S Madathil, C McGhee, K Mcgee, A Neal, A Newton Cox, J Nyaboko, D Parekh, Z Peterkin, H Qureshi, B Rangelov, L Ratcliffe, E Sapey, J Short, T Soulsby, R Steeds, J Stockley, Z Suleiman, T Thompson, M Ventura, S Walder, C Welch, D Wilson, S Yasmin, K P Yip

**University Hospitals of Derby and Burton**

P Beckett (PI) C Dickens, U Nanda

**University Hospitals of Leicester NHS Trust & University of Leicester**

C E Brightling (CI), R A Evans (PI), M Aljaroof, N Armstrong, H Arnold, H Aung, M Bakali, M Bakau, M Baldwin, M Bingham, M Bourne, C Bourne, N Brunskill, P Cairns, L Carr, A Charalambou, C Christie, M J Davies, S Diver, S Edwards, C Edwardson, O Elneima, H Evans, J Finch, S Glover, N Goodman, B Gootpu, N J Greening, B Guillen-Guio, K Hadley, P Haldar, B Hargadon, V C Harris, L Houchen-Wolloff, W Ibrahim, L Ingram, K Khunti, A Lea, D Lee, D Lozano-Rojas, G P McCann, H J C McAuley, P McCourt, T Mcnally, G Mills, A Moss, W Monteiro, K Ntotsis, M Pareek, S Parker, A Rowland, A Prickett, I N Qureshi, R Russell, N Samani, M Sereno, M Sharma, A Shikotra, S Siddiqui, A Singapuri, S J Singh, J Skeemer, M Soares, E Stringer, T Thornton, M Tobin, E Turner, L V Wain, T J C Ward, F Woodhead, J Wormleighton, T Yates, A Yousuf,

**University Hospital Southampton NHS Foundation Trust & University of Southampton**

M G Jones (PI), C Childs, R Djukanovic, S Fletcher, M Harvey, E Marouzet, B Marshall, R Samuel, T Sass, T Wallis, H Wheeler

**Whittington Health NHS**

R Dharmagunawardena (PI), E Bright, P Crisp, M Stern

**Wirral University Teaching Hospital**

A Wight (PI), L Bailey, A Reddington

**Wrightington Wigan and Leigh NHS trust**

A Ashish (PI), J Cooper, E Robinson

**Yeovil District Hospital NHS Foundation Trust**

A Broadley (PI)

**York & Scarborough NHS Foundation Trust**

K Howard (PI), L Barman, C Brookes, K Elliott. L Griffiths, Z Guy, D Ionita, H Redfearn, C Sarginson

A Turnbull

**Health and Care Research Wales**

Y Ellis

**London School of Hygiene & Tropical Medicine (LSHTM)**

M Marks, A Briggs

**NIHR Office for Clinical Research Infrastructure**

K Holmes

**Patient Public Involvement Leads**

Asthma UK and British Lung Foundation Partnership - K Poinasamy, S Walker

**Royal Surrey NHS Foundation Trust**

M Halling-Brown

**South London and Maudsley NHS Foundation Trust & Kings College London**

G Breen, M Hotopf

**Swansea University & Swansea Welsh Network**

K Lewis, N Williams

**Appendix B: Detail on Statistical Methods**

**EQ5D-5L Utility Index Scores**

EQ5D utility scores $(U)$ (higher score indicates a better quality of life) were transformed to disutility scores $\left( D \right)$ (higher score indicates worse quality of life) using a simple linear transformation $D=1-U$ to give a distribution of values with a right skew which is more easily amenable for statistical analysis. Then, in order to examine the potential effects of the health care pathway covariates on the EQ5D disutility at 12-months while controlling for potential differences in baseline demographic and clinical risk factors, a generalised linear mixed model (GLMM) model of the form:

|  | $ln \left( E\left[ D \right] \right) =X\beta+Y\gamma+\upsilon$ |  |
| --- | --- | --- |

was constructed, assuming a gamma distribution of disutility and a long link. $D$ is the EQ5D disutility index score, X are the covariates to be adjusted for, with coefficients $\beta$, $Y$ are the indicators of health care pathway, with coefficients $\gamma$, and $\upsilon$ is a random intercept at the level of the hospital. We run our statistical analysis in R and use the *glmmTMB* package (Brooks et al, 2017).

**Healthcare Resource Use and Associated Costs**

In order to examine the potential effect of the health care pathway covariates on the accumulated healthcare costs at 12 months post-discharge while controlling for potential differences in baseline demographic and clinical risk factors we construct a GLM model for cost with a Gamma distribution and log link:

|  | $ln \left( E\left[ C \right] \right) =X\beta+Y\gamma+\upsilon$ |  |
| --- | --- | --- |

where $C$ is the health care costs and the right hand side is as previously specified for disutility.

Appendix C – Supplementary Tables & Figures

**Table S1
Healthcare Resource items and associated unit costs for Health Service perspective**

| Healthcare Resource | Unit Cost (£) | Source |
| --- | --- | --- |
| *Rehabilitation* |  |  |
| - Initial assessment | 123 | NSHSC |
| - 10 sessions | 930 | NSHSC |
| *Mental Health Services* |  |  |
| - Post ICU psychology service | 507 | NSHSC |
| - Psychiatric liaison service (1.3 sessions) | 352 | NSHSC |
| - Improving Access to Psychological Therapies (7.5 sessions) | 990 | PSSRU |
| - Acute Hospital Clinical Health Psychology service | 155 | NSHSC |
| - Community Mental Health Team (adult) (8.7 sessions) | 2,047 | NSHSC |
| - Community Mental Health Team (older adult) (8.7 sessions) | 2,056 | NSHSC |
| - Mental health Crisis Resolution and Home Treatment service | 354 | NSHSC |
| - In patient mental health service | 632 | NSHSC |
| - Counselling services (Third sector or primary care based) | 279 | NSHSC |
| - Private providers of psychological therapy | 38 | PSSRU |
| *Basic Investigations* |  |  |
| - Chest X-Ray | 32 | SoECAT |
| - Electrocardiogram | 27 | SoECAT |
| *Blood tests* |  |  |
| - FBC | 12 | SoECAT |
| - U&Es | 8 | SoECAT |
| - eGFR | 12 | median |
| - LFTs | 12 | SoECAT |
| - CRP | 11 | SoECAT |
| - Bone profile | 8 | SoECAT |
| - 25-Hydroxyvitamin D (25-OH) | 12 | median |
| - BNP/Pro-NT BNP | 35 | SoECAT |
| - Troponin | 23 | SoECAT |
| - D Dimer | 15 | SoECAT |
| - Fibrinogen | 10 | SoECAT |
| - INR | 11 | SoECAT |
| - Ferritin | 11 | SoECAT |
| - HbA1C | 8 | SoECAT |
| - Lipid profile (non-fasting) | 12 | SoECAT |
| - SARS-Cov-2 Serology | 46 | SoECAT |
| *Respiratory Investigations and Procedures* |  |  |
| - Spirometry | 22 | SoECAT |
| - Full pulmonary function tests | 72 | NSHSC |
| - Fractional exhaled nitric oxide | 72 | NSHSC |
| - Nijmegen questionnaire | 22 | SoECAT |
| - Incremental shuttle walk test (ISWT) | 207 | NSHSC |
| - Six-minute walk test | 207 | NSHSC |
| - Cardio-pulmonary exercise test | 234 | NSHSC |
| - CT chest with contrast | 238 | SoECAT |
| - HRCT | 157 | SoECAT |
| - CTPA | 157 | SoECAT |
| - Pulmonary perfusion scan | 269 | NSHSC |
| *Cardiac Investigations and Procedures* |  |  |
| - Cardiac MRI | 374 | SoECAT |
| - Echocardiogram | 27 | SoECAT |
| - 24/48 hrs tape | 102 | SoECAT |
| - Right heart catheterisation | 258 | NSHSC |
| - Coronary angiogram/CT angiogram | 157 | SoECAT |
| - Cardiac perfusion scan | 344 | NSHSC |
| *Haematological Investigations and Procedures* |  |  |
| - Thyroid function tests | 30 | SoECAT |
| - Blood borne viruses screen | 30 | median |
| - Haematinics | 30 | median |
| *Renal Investigations and Procedures* |  |  |
| - USS abdomen | 59 | SoECAT |
| - USS Kidneys | 59 | SoECAT |
| - CT abdomen/pelvis | 157 | SoECAT |
| - Urinalysis | 22 | SoECAT |
| - Urine albumin: creatinine ratio | 22 | SoECAT |
| - Urine protein: creatinine ratio | 22 | SoECAT |
| *Immunological Investigations and Procedures* |  |  |
| - Complements factors | 23 | SoECAT |
| - Immunoglobulins | 23 | SoECAT |
| - Rheumatoid factor/Anti-CCP | 23 | SoECAT |
| *Neurology/Mental health Investigations and Procedures* |  |  |
| - CT brain | 157 | SoECAT |
| - MRI brain | 374 | SoECAT |
| - Nerve conduction studies | 100 | NSHSC |
| - Sleep Condition Indicator | 22 | SoECAT |
| *Emergency visit ITU/HDU admission* |  |  |
| - Short stay | 827 | PSSRU |
| - Long stay (>= 2 days) | 3,627 | PSSRU |
| Outpatient visit | 137 | PSSRU |
| Contacts with GP for a clinical review | 39 | PSSRU |

NSHSC = National Schedule of NHS Costs; PSSRU = Personal Social Services Research Unit; SoECAT = Schedule of Events Cost Attribution Tool; FBC - Full Blood Count; URES - Urea and Electrolytes; eGFR - Estimated Glomerular Filtration Rate; LFTs - Liver Function Tests; CRP - C-Reactive Protein; Pro-NT BNP - N-terminal pro b-type Natriuretic Peptide; INR - International Normalised Ratio; HbA1c - Haemoglobin A1c; SARS-CoV-2 - Severe Acute Respiratory Syndrome Coronavirus 2; ISWT - Incremental Shuttle Walk Test; CT – Computed Tomography; HRCT - High-Resolution Computed Tomography; CTPA - Computed Tomography Pulmonary Angiography; MRI - Magnetic Resonance Imaging; USS - Ultrasound Scan; Anti-CCP - Anti-Cyclic Citrullinated Peptide; ITU - Intensive Therapy Unit; HDU - High Dependency Unit; ; GP - General Practitioner.

**Table S2
EQ5D Utility scores by patient perceived recovery and newly diagnosed conditions**

| **Health State** | **Unadjusted mean (SD/n)** | **Adjusted mean (SE)** |
| --- | --- | --- |
| Feel fully recovered with no NDC | 0.87 (0.18 / 339) | 0.89 (0.007) |
| Feel fully recovered with NDC | 0.78 (0.21 / 139) | 0.84 (0.011) |
| Not recovered no NDC | 0.69 (0.25 / 592) | 0.76 (0.011) |
| Not recovered with NDC | 0.57 (0.28 / 475) | 0.66 (0.016) |

*Unadjusted scores based on observations, adjusted scores based on regression analyses holding all other covariates at mean values

**Table S3
Gamma distributed log-link GLM for EQ5D disutility at 5 and 12 months**

|  |  |  |  | 95% CI | |
| --- | --- | --- | --- | --- | --- |
|  | Estimate | SE | p-value | Lower | Upper |
| Intercept | 0.208 | 0.208 | 0.317 | -0.200 | 0.617 |
| Female | 0.237 | 0.062 | 0.000 | 0.115 | 0.359 |
| Pre-COVID Utility Index Summary Score | -2.136 | 0.156 | 0.000 | -2.441 | -1.830 |
| Index of Multiple Deprivation quintile |  |  |  |  |  |
| - 2 | 0.113 | 0.088 | 0.195 | -0.058 | 0.285 |
| - 3 | 0.060 | 0.092 | 0.510 | -0.119 | 0.240 |
| - 4 | -0.007 | 0.093 | 0.937 | -0.190 | 0.175 |
| - 5 - least deprived | -0.318 | 0.091 | 0.000 | -0.496 | -0.140 |
| Age at admission (years) |  |  |  |  |  |
| - <30 | -0.173 | 0.254 | 0.496 | -0.672 | 0.325 |
| - 30–39 | -0.212 | 0.135 | 0.117 | -0.478 | 0.053 |
| - 40–49 | -0.105 | 0.100 | 0.294 | -0.301 | 0.091 |
| - 60–69 | -0.109 | 0.073 | 0.138 | -0.252 | 0.035 |
| - 70–79 | -0.252 | 0.093 | 0.007 | -0.435 | -0.070 |
| - 80+ | 0.093 | 0.172 | 0.587 | -0.244 | 0.431 |
| WHO respiratory support class |  |  |  |  |  |
| - 5 | -0.138 | 0.087 | 0.111 | -0.308 | 0.032 |
| - 6 | -0.069 | 0.099 | 0.487 | -0.263 | 0.125 |
| - 7–9 | 0.370 | 0.100 | 0.000 | 0.175 | 0.566 |
| Baseline comorbidities |  |  |  |  |  |
| - Cardiac | 0.065 | 0.064 | 0.310 | -0.060 | 0.189 |
| - Respiratory | 0.180 | 0.065 | 0.005 | 0.053 | 0.307 |
| - Gastrointestinal | 0.055 | 0.085 | 0.519 | -0.112 | 0.221 |
| - Neurological and psychiatric | 0.281 | 0.080 | 0.000 | 0.123 | 0.438 |
| - Rheumatological | 0.249 | 0.092 | 0.007 | 0.069 | 0.430 |
| - Metabolic/Endocrine/Renal | -0.019 | 0.091 | 0.832 | -0.198 | 0.159 |
| - Malignancy/Haematological | 0.074 | 0.122 | 0.544 | -0.165 | 0.313 |
| Ethnicity |  |  |  |  |  |
| - South Asian | -0.115 | 0.109 | 0.295 | -0.329 | 0.100 |
| - Black | -0.101 | 0.113 | 0.371 | -0.323 | 0.121 |
| - Mixed | -0.190 | 0.201 | 0.344 | -0.583 | 0.203 |
| - Other | -0.151 | 0.156 | 0.332 | -0.456 | 0.154 |
| BMI >= 30kg/m^2^ | 0.195 | 0.060 | 0.001 | 0.078 | 0.312 |
| Days since discharge | 0.000 | 0.000 | 0.583 | 0.000 | 0.000 |
| Healthcare pathway |  |  |  |  |  |
| - Access and assessment | -0.223 | 0.097 | 0.022 | -0.414 | -0.032 |
| - Rehabilitation services | -0.178 | 0.065 | 0.006 | -0.306 | -0.051 |
| - Mental health services | 0.066 | 0.063 | 0.293 | -0.057 | 0.189 |
| - All patients offered services | 0.069 | 0.067 | 0.305 | -0.063 | 0.200 |

**Table S4
Gamma distributed log-link GLM for healthcare costs in 12-months post hospitalisation**

|  |  |  |  | 95% CI | |
| --- | --- | --- | --- | --- | --- |
|  | Estimate | SE | p-value | Lower | Upper |
| Intercept | 7.156 | 0.389 | 0.000 | 6.393 | 7.918 |
| Female | 0.236 | 0.079 | 0.003 | 0.082 | 0.390 |
| Pre-COVID Utility Index Summary Score | -0.417 | 0.199 | 0.036 | -0.807 | -0.028 |
| Index of Multiple Deprivation quintile |  |  |  |  |  |
| - 2 | -0.102 | 0.112 | 0.362 | -0.321 | 0.117 |
| - 3 | -0.274 | 0.116 | 0.018 | -0.501 | -0.047 |
| - 4 | -0.339 | 0.116 | 0.003 | -0.566 | -0.112 |
| - 5 - least deprived | -0.350 | 0.117 | 0.003 | -0.580 | -0.120 |
| Age at admission (years) |  |  |  |  |  |
| - <30 | -0.165 | 0.290 | 0.571 | -0.734 | 0.405 |
| - 30–39 | -0.361 | 0.167 | 0.030 | -0.688 | -0.035 |
| - 40–49 | -0.057 | 0.123 | 0.646 | -0.298 | 0.185 |
| - 60–69 | -0.232 | 0.089 | 0.010 | -0.407 | -0.057 |
| - 70–79 | -0.016 | 0.116 | 0.892 | -0.244 | 0.212 |
| - 80+ | 0.037 | 0.208 | 0.857 | -0.370 | 0.445 |
| WHO respiratory support class |  |  |  |  |  |
| - 5 | -0.237 | 0.110 | 0.032 | -0.453 | -0.021 |
| - 6 | -0.050 | 0.122 | 0.682 | -0.288 | 0.189 |
| - 7–9 | 0.510 | 0.128 | 0.000 | 0.259 | 0.761 |
| Baseline comorbidities |  |  |  |  |  |
| - Cardiac | 0.008 | 0.077 | 0.918 | -0.142 | 0.158 |
| - Respiratory | 0.520 | 0.080 | 0.000 | 0.363 | 0.678 |
| - Gastrointestinal | 0.096 | 0.106 | 0.364 | -0.111 | 0.303 |
| - Neurological and psychiatric | 0.189 | 0.101 | 0.060 | -0.008 | 0.387 |
| - Rheumatological | -0.089 | 0.121 | 0.464 | -0.326 | 0.149 |
| - Metabolic/Endocrine/Renal | 0.102 | 0.115 | 0.374 | -0.124 | 0.328 |
| - Malignancy/Haematological | 0.307 | 0.148 | 0.038 | 0.017 | 0.597 |
| Ethnicity |  |  |  |  |  |
| - South Asian | -0.247 | 0.145 | 0.089 | -0.532 | 0.037 |
| - Black | -0.037 | 0.242 | 0.878 | -0.511 | 0.436 |
| - Mixed | -0.214 | 0.191 | 0.264 | -0.589 | 0.161 |
| - Other | -0.142 | 0.077 | 0.063 | -0.293 | 0.008 |
|  |  |  |  |  |  |
| BMI >= 30kg/m^2^ | -0.239 | 0.129 | 0.065 | -0.493 | 0.014 |
| Healthcare pathway |  |  |  |  |  |
| - Access and assessment | -0.049 | 0.358 | 0.892 | -0.750 | 0.653 |
| - Rehabilitation services | 0.034 | 0.299 | 0.909 | -0.551 | 0.619 |
| - Mental health services | -0.122 | 0.301 | 0.684 | -0.712 | 0.467 |
| - All patients offered service | 0.312 | 0.310 | 0.313 | -0.295 | 0.919 |

**Table S5
Estimated QALYs at one year and costs for each of the unique 11 health care pathways available at PHOSP-COVID sites***The presence of each of the four main effects (assessment, rehabilitation, mental health, service offered to all) are shown in the first four columns. The last column of the table shows the label for that pathway*

| Comprehensive services | | | All patients invited to attend clinic?  No (selected)  Yes (all) |  | | | |  |
| --- | --- | --- | --- | --- | --- | --- | --- | --- |
| Assessment  (A) | Rehabilitation  (R) | Mental health (MH) |  | QALY | SE | Cost (£) | SE | label |
| No | No | No | No | 0.725 | 0.026 | 755 | 276 | 0. Low-selected |
| No | No | No | Yes | 0.706 | 0.032 | 1032 | 479 | 1. Low-all |
| No | No | Yes | Yes | 0.686 | 0.038 | 914 | 484 | 2. MH-all |
| No | Yes | No | No | 0.770 | 0.026 | 782 | 392 | 3.R-selected |
| Yes | No | No | No | 0.780 | 0.013 | 720 | 237 | 4.A-selected |
| Yes | No | No | Yes | 0.764 | 0.014 | 983 | 305 | 5.A-all |
| Yes | No | Yes | No | 0.765 | 0.016 | 637 | 252 | 6.A-MH-selected |
| Yes | No | Yes | Yes | 0.748 | 0.018 | 870 | 286 | 7.A-MH-all |
| Yes | Yes | No | No | 0.816 | 0.015 | 744 | 300 | 8.A-R-selected |
| Yes | Yes | No | Yes | 0.803 | 0.016 | 659 | 297 | 9.A-R-all |
| Yes | Yes | Yes | Yes | 0.789 | 0.012 | 900 | 269 | 10.A-R-MH-all |

**Table S6
Gamma distributed log-link GLM for EQ5D (weighted)**

|  |  |  |  | | 95% CI | | |
| --- | --- | --- | --- | --- | --- | --- | --- |
|  | Estimate | SE | p-value | | Lower | | Upper |
| Intercept | 0.208 | 0.208 | | 0.317 | | -0.200 | 0.617 |
| Female | 0.237 | 0.062 | | 0.000 | | 0.115 | 0.359 |
| Pre-COVID Utility Index Summary Score | -2.136 | 0.156 | | 0.000 | | -2.441 | -1.830 |
| Index of Multiple Deprivation quintile |  |  | |  | |  |  |
| - 2 | 0.113 | 0.088 | | 0.195 | | -0.058 | 0.285 |
| - 3 | 0.060 | 0.092 | | 0.510 | | -0.119 | 0.240 |
| - 4 | -0.007 | 0.093 | | 0.937 | | -0.190 | 0.175 |
| - 5 - least deprived | -0.318 | 0.091 | | 0.000 | | -0.496 | -0.140 |
| Age at admission (years) |  |  | |  | |  |  |
| - <30 | -0.173 | 0.254 | | 0.496 | | -0.672 | 0.325 |
| - 30–39 | -0.212 | 0.135 | | 0.117 | | -0.478 | 0.053 |
| - 40–49 | -0.105 | 0.100 | | 0.294 | | -0.301 | 0.091 |
| - 60–69 | -0.109 | 0.073 | | 0.138 | | -0.252 | 0.035 |
| - 70–79 | -0.252 | 0.093 | | 0.007 | | -0.435 | -0.070 |
| - 80+ | 0.093 | 0.172 | | 0.587 | | -0.244 | 0.431 |
| WHO respiratory support class |  |  | |  | |  |  |
| - 5 | -0.138 | 0.087 | | 0.111 | | -0.308 | 0.032 |
| - 6 | -0.069 | 0.099 | | 0.487 | | -0.263 | 0.125 |
| - 7–9 | 0.370 | 0.100 | | 0.000 | | 0.175 | 0.566 |
| Baseline comorbidities |  |  | |  | |  |  |
| - Cardiac | 0.065 | 0.064 | | 0.310 | | -0.060 | 0.189 |
| - Respiratory | 0.180 | 0.065 | | 0.005 | | 0.053 | 0.307 |
| - Gastrointestinal | 0.055 | 0.085 | | 0.519 | | -0.112 | 0.221 |
| - Neurological and psychiatric | 0.281 | 0.080 | | 0.000 | | 0.123 | 0.438 |
| - Rheumatological | 0.249 | 0.092 | | 0.007 | | 0.069 | 0.430 |
| - Metabolic/Endocrine/Renal | -0.019 | 0.091 | | 0.832 | | -0.198 | 0.159 |
| - Malignancy/Haematological | 0.074 | 0.122 | | 0.544 | | -0.165 | 0.313 |
| Ethnicity |  |  | |  | |  |  |
| - South Asian | -0.115 | 0.109 | | 0.295 | | -0.329 | 0.100 |
| - Black | -0.101 | 0.113 | | 0.371 | | -0.323 | 0.121 |
| - Mixed | -0.190 | 0.201 | | 0.344 | | -0.583 | 0.203 |
| - Other | -0.151 | 0.156 | | 0.332 | | -0.456 | 0.154 |
| BMI >= 30kg/m^2^ | 0.195 | 0.060 | | 0.001 | | 0.078 | 0.312 |
| Days since discharge | 0.000 | 0.000 | | 0.583 | | 0.000 | 0.000 |
| Healthcare pathway |  |  | |  | |  |  |
| - Access and assessment | -0.223 | 0.097 | | 0.022 | | -0.414 | -0.032 |
| - Rehabilitation services | -0.178 | 0.065 | | 0.006 | | -0.306 | -0.051 |
| - Mental health services | 0.066 | 0.063 | | 0.293 | | -0.057 | 0.189 |
| - All patients offered services | 0.069 | 0.067 | | 0.305 | | -0.063 | 0.200 |

**Table S7
Gamma distributed log-link GLM for healthcare costs in 12-months post hospitalisation (weighted)**

|  |  |  |  | 95% CI | |
| --- | --- | --- | --- | --- | --- |
|  | Estimate | SE | p-value | Lower | Upper |
| Intercept | 6.822 | 0.400 | 0.000 | 6.039 | 7.605 |
| Female | 0.083 | 0.076 | 0.274 | -0.066 | 0.232 |
| Pre-COVID Utility Index Summary Score | -0.348 | 0.177 | 0.049 | -0.695 | -0.002 |
| Index of Multiple Deprivation quintile |  |  |  |  |  |
| - 2 | -0.065 | 0.096 | 0.497 | -0.254 | 0.123 |
| - 3 | 0.039 | 0.109 | 0.718 | -0.174 | 0.252 |
| - 4 | -0.001 | 0.111 | 0.990 | -0.219 | 0.216 |
| - 5 - least deprived | -0.033 | 0.120 | 0.786 | -0.267 | 0.202 |
| Age at admission (years) |  |  |  |  |  |
| - <30 | -0.734 | 0.142 | 0.000 | -1.013 | -0.456 |
| - 30–39 | -0.398 | 0.142 | 0.005 | -0.676 | -0.119 |
| - 40–49 | 0.124 | 0.132 | 0.346 | -0.134 | 0.383 |
| - 60–69 | -0.234 | 0.119 | 0.050 | -0.467 | 0.000 |
| - 70–79 | -0.108 | 0.120 | 0.371 | -0.343 | 0.128 |
| - 80+ | 0.076 | 0.133 | 0.566 | -0.184 | 0.337 |
| WHO respiratory support class |  |  |  |  |  |
| - 5 | -0.002 | 0.085 | 0.979 | -0.170 | 0.165 |
| - 6 | 0.166 | 0.104 | 0.111 | -0.038 | 0.371 |
| - 7–9 | 0.749 | 0.200 | 0.000 | 0.357 | 1.141 |
| Baseline comorbidities |  |  |  |  |  |
| - Cardiac | -0.116 | 0.083 | 0.165 | -0.279 | 0.048 |
| - Respiratory | 0.691 | 0.081 | 0.000 | 0.533 | 0.850 |
| - Gastrointestinal | 0.108 | 0.100 | 0.279 | -0.088 | 0.304 |
| - Neurological and psychiatric | -0.013 | 0.104 | 0.899 | -0.218 | 0.191 |
| - Rheumatological | 0.001 | 0.119 | 0.991 | -0.231 | 0.234 |
| - Metabolic/Endocrine/Renal | 0.039 | 0.111 | 0.728 | -0.180 | 0.257 |
| - Malignancy/Haematological | 0.342 | 0.153 | 0.025 | 0.043 | 0.641 |
| Ethnicity |  |  |  |  |  |
| - South Asian | -0.385 | 0.112 | 0.001 | -0.604 | -0.166 |
| - Black | 0.231 | 0.136 | 0.089 | -0.035 | 0.497 |
| - Mixed | -0.082 | 0.234 | 0.727 | -0.539 | 0.376 |
| - Other | -0.033 | 0.226 | 0.884 | -0.477 | 0.410 |
| BMI >= 30kg/m^2^ | 0.009 | 0.073 | 0.896 | -0.133 | 0.152 |
| Healthcare pathway |  |  |  |  |  |
| - Access and assessment | 0.036 | 0.394 | 0.928 | -0.736 | 0.807 |
| - Rehabilitation services | -0.181 | 0.315 | 0.565 | -0.798 | 0.436 |
| - Mental health services | -0.228 | 0.317 | 0.473 | -0.850 | 0.394 |
| - All patients offered service | 0.415 | 0.333 | 0.212 | -0.237 | 1.066 |

**Figure S1
Forest plot of the impact of each health care pathway in PHOSP-COVID on Cost compared to the lowest service offering**

*Lowest service included both no follow-up service and no comprehensive element of the service. A = Assessment, MH = Mental Health Services, R = Rehabilitation, all = all patients potentially could access the service, selected = only a pre-specified sub-group could access the service
